# Dermatofibrosarcoma Protuberans Found Within Procedural Scars: A Retrospective Review at a Tertiary Referral Cancer Center

**DOI:** 10.7759/cureus.10286

**Published:** 2020-09-07

**Authors:** Ravi C Patel, Christopher Downing, Caitlin Robinson, Roland Bassett, Christina L Roland, Naveen Garg, Philip R Cohen, Deborah F MacFarlane

**Affiliations:** 1 Dermatology, MD Anderson Cancer Center, Houston, USA; 2 Dermatology, Cypress Dermatology, Cypress, USA; 3 Dermatology, Colorado Springs Dermatology Clinic, Colorado Springs, USA; 4 Biostatistics, MD Anderson Cancer Center, Houston, USA; 5 Surgical Oncology, MD Anderson Cancer Center, Houston, USA; 6 Radiology, MD Anderson Cancer Center, Houston, USA; 7 Dermatology, San Diego Family Dermatology, National City, USA

**Keywords:** dermatofibrosarcoma protuberans, dfsp, procedural scars

## Abstract

Background: Surgical scars are not a well-known risk factor for the development of dermatofibrosarcoma protuberans (DFSP). However, DFSP can arise within a surgical scar.

Objective: This study determined the number of DFSP found within scars from prior surgical procedures at a tertiary academic cancer center.

Methods: A retrospective data analysis was performed of all patients with biopsy-proven DFSP from January 2000 to April 2018 at MD Anderson Cancer Center (MDACC), a tertiary referral cancer center. Chart review was performed, and data were recorded for gender, race, and age of patients. We also recorded the site, location, and size of the DFSP and whether the patients had a history of prior surgery at the DFSP site. All patients had a pathologic diagnosis of DFSP at MDACC. Patients were selected from the pathology database at MDACC using the keywords “DFSP” or “dermatofibrosarcoma protuberans”. A total of 458 patients were identified; however, 94 patients were excluded from the study because they were only referred to MDACC with a confirmed diagnosis of DFSP and were not seen as patients by a MDACC physician.

Results: Of the remaining 364 patients, 37 patients (10.1%) had either a history of a benign neoplasm or an inflammatory disease that had been evaluated or treated by either punch biopsy, shave biopsy, or minor excision at the site of DFSP (8.5%, 31 patients) or a DFSP arising within a major surgical procedural scar (1.6%, six patients). The surgical sites identified were the abdomen (four patients) and the groin (two patients). Three of the patients with a major surgical scar DFSP had prior laparoscopic surgery at the site.

Conclusions: DFSPs occur at surgical scars. The development of surgical scar DFSP in 10% of our patients prompts us to postulate that the neoplasm may be associated with the malignant transformation of these scars.

## Introduction

Dermatofibrosarcoma protuberans (DFSP) is a rare subcutaneous and/or dermal tumor that is locally aggressive with frequent recurrence. However, there is less local recurrence when DFSP is treated with Mohs micrographic surgery [1]. Moreover, metastases are rare. It has an annual incidence in the United States of approximately 4.5 per million [2].

The tumor is typically diagnosed in young to mid-aged patients. The incidence in blacks is almost two times higher than in whites. In addition, a poorer survival is associated with increased age, male gender, tumors located on the limbs and head, and black race [3].

DFSP is most commonly found on the trunk (42%) followed by the lower extremities (21%) and upper extremities (21%). The head and neck region is affected in 16% of individuals. Patients typically present with a slow-growing, irregular dermal plaque that is either skin-colored or slightly yellow, pink, or brown. Occasionally the tumor presents as firm erythematous nodules with irregular borders [3].

The pathogenesis of DFSP may be multifactorial. Trauma, from either surgical, immunization, or burn scars, is an inciting factor for DFSP. In addition, more than 90% of DFSPs present with a chromosomal translocation involving chromosomes 17q22 and 22q13, with a fusion of the genes collagen, type1, alpha1 (COL1A1) and platelet-derived growth factor subunit B (PDGFB) [4-8].

Surgery, including laparoscopic, arthroscopic, and minimally invasive procedures, is prevalent in the USA [9-12]. We evaluated two elderly women who presented with an abdominal DFSP that developed in a laparoscopic surgery scar from a previous gynecological procedure. Our observation of these two patients with scar-related DFSP prompted us to investigate whether there is any association between surgical scars and the subsequent development of DFSP in these sites.

## Materials and methods

This retrospective analysis was an institutional based, observational study at MD Anderson Cancer Center (MDACC) in Houston, Texas. Cases from January 2000 to April 2018 were retrieved from in the pathology database at MDACC; the diagnosis of DFSP from any site of the body was searched. A total of 458 patient medical record numbers were obtained. Patient charts were analyzed for the following: date of birth, race, gender, confirmation of DFSP diagnosis, body location of DFSP, and date of DFSP diagnosis. In addition, the patient’s notes were reviewed to determine if the individual had a history of surgery in the same site as the DFSP. If the information was available, the type and date of original surgery was obtained. The diagnosis date was defined as the date when the DFSP biopsy was performed. Ninety-four patients with a DFSP diagnosis in our database had records and pathology specimens sent to MDACC but were not seen as patients by a MDACC physician; therefore, these individuals were not included in our analysis.

## Results

A total of 458 patients with DFSP were found within our patient database from January 2000 to April 2018. However, 94 individuals who were never seen by a MDACC physician were excluded.

At the time of DFSP diagnosis, age ranged from 13 to 73 (mean, 43 years, median, 40 years). DFSP were observed in 194 women (53.4%) and 170 men (46.7%).

Most of the 364 patients were Caucasian (236, 64.8%). DFSP were also observed in Black (55, 15.1%), Hispanic (36, 9.9%), and Asian (17, 4.7%) patients. The race was not available for 20 (5.5%) of the patients.

The tumor location for the 364 patients is provided in Table 1. The DFSP was most commonly located on the torso (40.4%). They also occurred on the extremities (28%) and the head and neck (19.2%). Less often, they were located on the suprapubic area or buttocks (12.4%).

**Table 1:** Location of dermatofibrosarcoma protuberans

Location	Number	Percent
Torso		
Abdomen	51	14.0
Chest	49	13.5
Back	47	12.9
Total	147	40.4
Extremities		
Arm	55	15.1
Leg	47	12.9
Total	102	28.0
Head and Neck	70	19.2
Total	70	19.2
Suprapubic and Buttock		
Suprapubic	35	9.6
Buttock	10	2.8
Total	45	12.4
Overall Total	364	100.0

**Figure 1:**
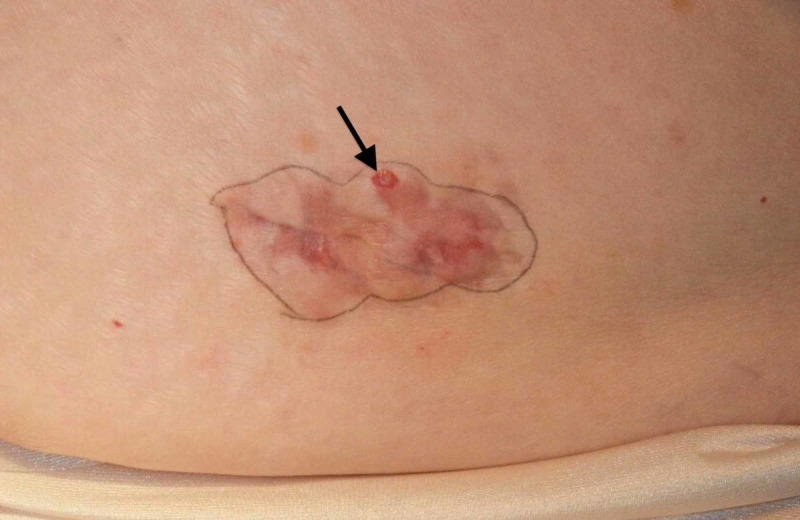
Laparoscopy scar-associated dermatofibrosarcoma protuberans A 44-year-old woman had laparoscopic surgery for endometrioma and endometriosis. She subsequently developed an 8 x 2.5 centimeter dermatofibrosarcoma protuberans (black arrow) in her surgical scar on the left suprapubic area.

**Figure 2:**
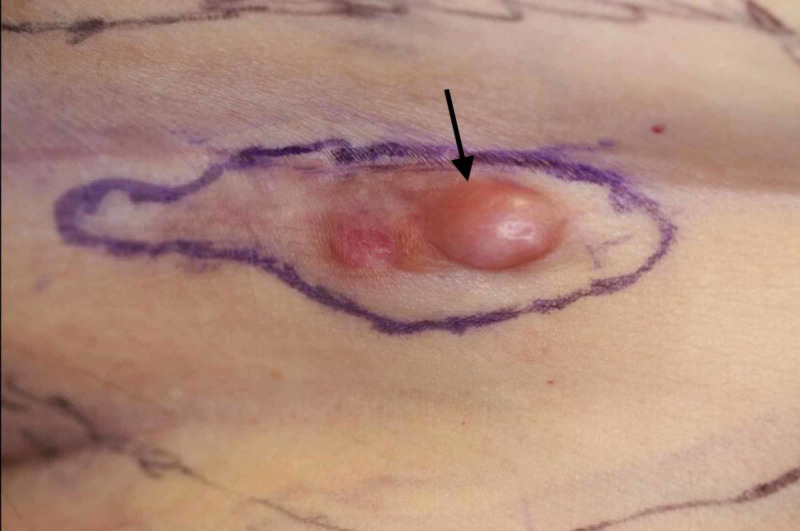
Scar related dermatofibrosarcoma protuberans at a prior laparoscopy site A 75-year-old woman who had a bilateral laparoscopic salpingo-oophorectomy. She subsequently developed an ill-defined red dermal plaque (black arrow) in her surgical scar on the left abdomen. A biopsy of the lesion confirmed the diagnosis of dermatofibrosarcoma protuberans within the scar.

Six patients (1.7%)--five women and one man--developed DFSP at the site of a previous major procedural surgical scar. The characteristics of patients with a DFSP found within an existing major procedural surgical scar are summarized in Table 2. Tumors were found in the abdomen (four patients) and suprapubic region (two patients). Three (50%) of these patients with DFSP found within existing surgical scars were noted to have a previous laparoscopic surgery (Figure 1 and Figure 2).

**Table 2:** Characteristics of six patients with a DFSP arising within a major surgical procedural scar Abbreviations: Ca= Caucasian, DFSP= Dermatofibrosarcoma protuberans, M= Man, NS= Not stated, W= Woman, yr= years, >= greater than, <= less than a. The largest diameter in centimeters

Case	Age (yr) Race Gender	DFSP Location	Prior Surgery	Time between surgery and DFSP diagnosis	DFSP Size^a^
1	35 NS W	Abdomen	Cesarean section	<5 years	1.8
2	44 Ca W	Suprapubic	Laparoscopy for endometriosis and endometrioma	>10 years	8.0
3	45 Ca M	Suprapubic	Inguinal hernia repair	>10 years	3.9
4	45 Ca W	Abdomen	Laparoscopic cholecystectomy	>10 years	3.5
5	56 Ca W	Abdomen	Tubal ligation	> 10 years	NS
6	75 Ca W	Abdomen	Laparoscopic bilateral salpingo-oophorectomy	> 10 years	NS

Thirty-one (17 women and 14 men) patients developed a DFSP at the site of a prior surgical scar from a skin biopsy or minor excision. Most of the individuals were Caucasian (21 patients, 67.8%). The other patients were Black (four, 2.9%), Hispanic (three, 9.6%), or Asian (two, 6.4%). The race was not decided for one patient (3.2%).

The tumor location for the 31 patients who developed a DFSP at the site of a prior skin biopsy or minor excision is provided in Table 3. The DFSP was most commonly located on the torso (54.7%). They also occurred on the extremities (19.3%) and the head (19.3%). Less often, they were located on the suprapubic area (6.4%).

**Table 3:** Location of DFSP at skin biopsy or minor excision sites DFSP= Dermatofibrosarcoma protuberans

Location	Number	Percentage
Torso		
Back	7	22.5
Abdomen	5	16.1
Chest	5	16.1
Total	17	54.7
Extremities		
Arm	5	16.0
Leg	1	3.2
Total	6	19.3
Head		
Total	6	19.3
Suprapubic		
Total	2	6.4
Overall Total	31	100.0

The skin biopsy-related or minor excision-associated DFSP were most commonly at the site of epidermoid cyst removal (nine patients, 29.0%). The next most frequent benign tumor excision was a lipoma (four patients, 12.9%). The following lesions were each the DFSP site for one patient (3.2%): acrochordon, bone spur, calcinosis cutis, dermatofibroma, neurofibroma, nevus, and sarcoidosis. The associated excised lesion was not described for 11 patients (35.4%).

## Discussion

DFSPs develop at surgical scars. In our study, six patients had a major surgical procedure located either on the abdomen (four patients) or suprapubic region (two patients).

A benign neoplasm or inflammatory disease was either sampled or treated by shave biopsy, punch biopsy, or minor excision in 31 patients. However, the resulting scar either never healed as anticipated, or grew in size over time. Some of these individuals delayed evaluation of their DFSP since they were told by their physician that the lesion was benign.

There is a paucity of medical literature regarding DFSP developing in a surgical scar. Stivala et al. published a study of 59 DFSP patients. The DFSP arose in the previous surgical scars in five (8.4%) patients. The location of the scar was in the groin area (two patients), the left ankle (one patient), the back (one patient), and the scalp (one patient) [9].

Muller et al. reported a 40-year-old woman with a giant cell fibroblastoma, a juvenile form of DFSP, arising in a post-surgical scar. The patient underwent an endoscopic corrective procedure for an atrial septal defect and ten months later was noted to have a 15 x 15 mm dome-shaped papule that was originally thought to be a keloid. After another 14 months, the tumor was noted to be 20 x 45 mm and pathology confirmed a giant cell fibroblastoma [10].

Other reports describe DFSPs in non-surgical scars including vaccination scars and burn scars. McLelland and Chu described a DFSP arising within a bacilli Calmette-Guerin (BCG) vaccination scar in a 28-year-old man who had been vaccinated 15 years previously [11]. In our study population, a 38-year-old woman had a DFSP arise within a chicken pox scar.

Seo et al. reported a DFSP within a chronic burn scar in a 43-year-old Korean man who suffered a burn at eight years of age [12]. In a review of 115 patients with DFSP, 16.5% had a history of local trauma including surgical, burn, vaccination scar, contusion, fracture, or folliculitis [13].

Saeki et al. reported a 43-year-old man who had an appendectomy with subsequent drainage tube insertion. He was diagnosed with a DFSP at the surgical site 20 years later. Five years before the diagnosis of DFSP, the patient noticed a small papule overlying the scar that continued to slowly enlarge [14].

Local trauma, wound healing with subsequent scar formation, and antigenic stimulation or oncogenic transduction by inoculated infectious agents have been implicated in the pathogenesis of malignancy [15]. Trauma is a risk factor for development of DFSP in surgical scars, burn scars, and immunization scars [16]. Trauma has also been cited as a risk factor for development of DFSP within a tattoo [17].

We speculate that one possibility for the development of DFSPs in a surgical scar may be secondary to the malignant transformation of these scars. Other cancers such as squamous cell carcinoma or lung scar carcinoma can develop within scar tissue. It is reasonable to hypothesize chronic inflammation could lead to the malignant transformation of scars in DFSP.

To the best of our knowledge, this is the largest study to report a relationship between DFSP development and surgical scars. The study is limited in that it is a retrospective study from a single cohort of patients. Also, although a potential correlation between surgical scars and DFSP has been proposed, causality has not yet been established. Therefore, further research may be necessary to reinforce this correlation and determine causation.

## Conclusions

The pathogenesis of DFSP remains to be established. It is likely to be multifactorial. We observed a 10% incidence of DFSP at the site of a prior surgical scar. Secondary malignant transformation of scars may be one of the etiologic factors involved in the pathogenesis of DFSP.
